# Characterizing Tweet Volume and Content About Common Health Conditions Across Pennsylvania: Retrospective Analysis

**DOI:** 10.2196/10834

**Published:** 2018-12-06

**Authors:** Christopher Tufts, Daniel Polsky, Kevin G Volpp, Peter W Groeneveld, Lyle Ungar, Raina M Merchant, Arthur P Pelullo

**Affiliations:** 1 Center for Digital Health Penn Medicine Philadelphia, PA United States; 2 Leonard Davis Institute of Health Economics University of Pennsylvania Philadelphia, PA United States; 3 Center for Health Equity Research and Promotion University of Pennsylvania Philadelphia, PA United States; 4 Population Studies Center School of Arts and Science University of Pennsylvania Philadelphia, PA United States; 5 Department of Medical Ethics and Policy University of Pennsylvania Philadelphia, PA United States; 6 Philadelphia VA Medical Center Philadelphia, PA United States; 7 Department of General Internal Medicine University of Pennsylvania Philadelphia, PA United States; 8 Department of Computer and Information Science School of Engineering and Applied Science University of Pennsylvania Philadelphia, PA United States; 9 Department of Emergency Medicine University of Pennsylvania Philadelphia, PA United States

**Keywords:** Twitter messaging, disease, prevalence, public health surveillance, social media

## Abstract

**Background:**

Tweets can provide broad, real-time perspectives about health and medical diagnoses that can inform disease surveillance in geographic regions. Less is known, however, about how much individuals post about common health conditions or what they post about.

**Objective:**

We sought to collect and analyze tweets from 1 state about high prevalence health conditions and characterize the tweet volume and content.

**Methods:**

We collected 408,296,620 tweets originating in Pennsylvania from 2012-2015 and compared the prevalence of 14 common diseases to the frequency of disease mentions on Twitter. We identified and corrected bias induced due to variance in disease term specificity and used the machine learning approach of differential language analysis to determine the content (words and themes) most highly correlated with each disease.

**Results:**

Common disease terms were included in 226,802 tweets (174,381 tweets after disease term correction). Posts about breast cancer (39,156/174,381 messages, 22.45%; 306,127/12,702,379 prevalence, 2.41%) and diabetes (40,217/174,381 messages, 23.06%; 2,189,890/12,702,379 prevalence, 17.24%) were overrepresented on Twitter relative to disease prevalence, whereas hypertension (17,245/174,381 messages, 9.89%; 4,614,776/12,702,379 prevalence, 36.33%), chronic obstructive pulmonary disease (1648/174,381 messages, 0.95%; 1,083,627/12,702,379 prevalence, 8.53%), and heart disease (13,669/174,381 messages, 7.84%; 2,461,721/12,702,379 prevalence, 19.38%) were underrepresented. The content of messages also varied by disease. Personal experience messages accounted for 12.88% (578/4487) of prostate cancer tweets and 24.17% (4046/16,742) of asthma tweets. Awareness-themed tweets were more often about breast cancer (9139/39,156 messages, 23.34%) than asthma (1040/16,742 messages, 6.21%). Tweets about risk factors were more often about heart disease (1375/13,669 messages, 10.06%) than lymphoma (105/4927 messages, 2.13%).

**Conclusions:**

Twitter provides a window into the Web-based visibility of diseases and how the volume of Web-based content about diseases varies by condition. Further, the potential value in tweets is in the rich content they provide about individuals’ perspectives about diseases (eg, personal experiences, awareness, and risk factors) that are not otherwise easily captured through traditional surveys or administrative data.

## Introduction

Communities are increasingly identified as a driver of health, yet our ability to track changes in the health of communities has been limited by the nature of community-level data. These data are typically survey-based or derived from administrative health care claims. In both of these cases, delays in data availability can preclude timely interventions. Social media channels, like Twitter, offer a new opportunity to track regional health trends by observing health-related communication generated by the public and for the public [1-7].

There is an opportunity to determine how emerging digital data sources are complementary (ie, social media data have similar findings to traditional health data sources) and augmentative (ie, social media provides new real-time information about health not available in data collected through traditional means). To better quantify the value added by social media for public health surveillance, an understanding of how much data exist about different health conditions is needed. High prevalence conditions that affect much of a population may be underrepresented on the Web, whereas low prevalence conditions could be discussed more frequently on Twitter. Further, it is likely that there are different drivers (eg, disease morbidity and mortality, celebrity news, acuity, and stigma) that may influence the volume of Web-based health conversations.

To better characterize health-related tweet volume and content, we compared the volume of Twitter messages about common diseases with the prevalence of the disease determined from inpatient and outpatient claims. We then characterized the public perception of common diseases by identifying the content (words and themes) most frequently associated with each condition.

## Methods

### Context

This was a retrospective analysis of publicly available data about health conditions posted on Twitter in Pennsylvania. This study was approved by the University of Pennsylvania Institutional Review Board.

We collected tweets originating from Pennsylvania related to 5 of the top causes of death in the United States. The causes of death were then further divided into subcategories: heart disease (heart disease and hypertension), diabetes, stroke, cancer (breast, skin, lung, lymphoma, leukemia, prostate, pancreatic, and ovarian), and chronic lung disease (asthma, chronic obstructive pulmonary disease, COPD).

### Data Sources

#### Twitter Data

Twitter is a social media platform that allows users to send and receive short messages called “tweets.” At the time of data collection, tweets were limited to 140 characters; this limit was doubled to 280 characters in 2017. All tweets were collected via the Twitter Application Programming Interface (API) as described in Preotiuc-Pietro et al [8]. First, the Twitter Streaming API was used to collect a random 1% sample of public tweets from 2012-2015. This initial dataset was then filtered to contain only geolocated tweets or tweets originating from users with nonempty location fields in their profile. The county of origin of each tweet user was determined, and the dataset was filtered to obtain only tweets for users in Pennsylvania. To increase the sample size of tweets from the state, all unique user IDs were recorded, and the Twitter search API was used to extract timelines (each user’s prior 3200 tweets) filtered by timestamps ranging from 2012-2015.

#### Disease Keywords

The dataset analyzed was filtered for messages containing at least 1 keyword referencing a disease. The lexica of keywords (Multimedia Appendix 1) for each disease was derived from the Consumer Health Vocabulary [9] and supplemented by the authors of the study. The precision of the keyword filtering was estimated for each disease via a correction factor derived from a manual review of the tweets. The correction factor was then used to calculate corrected message counts.

#### Tweet Location

All tweets used in this analysis were classified as originating from a county in Pennsylvania. The tweets were mapped to a county using a combination of coordinates and the user-provided location field as per the method described in Schwartz et al [10]. For county mapping, we identified if coordinates were present with the tweet. If coordinates were present, these were used to identify the county of origin via the Google Maps API. For tweets without coordinates, we used the location field provided in the user’s profile to identify the county. When the field contained only a city or city nickname, it was mapped to a county as long as it met the following criteria: at least 90% of the population in all the cities with that name are in 1 specific city. For example, “Chicago” would get mapped to Chicago, Illinois, because greater than 90% of the population in all cities named “Chicago” in the United States are located in Chicago, Illinois. “Springfield” would not be mapped, as there are approximately 50 different regions named “Springfield” in the United States of similar population density. The same process in the previous step was used if the county name was listed without a specified state. Cities that were among the top 1000 English or Spanish nouns, verbs, and adjectives were not considered.

#### Deriving Topics About Individual Diseases

Utilizing all messages from the dataset, 200 topics (ie, groups of co-occurring words) were generated using the Mallet implementation of latent Dirichlet allocation (LDA). The input data for LDA were filtered to remove all disease keywords along with all words used by less than 5% of tweet authors.

The topic distribution of each message was then calculated as described in Schwartz et al [11]. The Pearson correlation between topic distribution and a binary label of whether or not the tweet contained the disease mentioned was calculated. All correlations were corrected for false discovery rate using the Benjamini-Hochberg procedure.

#### Organizing Topics into Themes

We created 10 themes by clustering the 200 LDA topics using nonnegative matrix factorization of the LDA topics derived from the messages. We identified the resulting clusters of topics as “themes.” The LDA topics specify the probability of each word given each topic. Nonnegative matrix factorization provides a weighted value indicating how much each topic, and hence each word in each topic, contributes to each theme. Theme distributions for each message were then calculated in the same manner as described previously for the topic distributions, using Bayes’ rule to compute p(theme|word). The resulting themes were manually labeled as follows: News, Research, Slang or Popular Culture Reference, Environment, Diagnosis and Survivorship, Treatment, Diet and Prevention, Awareness, Risk Factor, and Personal Experience.

### Statistical Analysis

#### Disease Prevalence

Outpatient and inpatient hospitalization claims were retrieved from 2013 and 2014 claims data from the Pennsylvania Health Care Cost Containment Council. Claims corresponding to each disease were identified using the primary and secondary diagnostic codes that were encoded via the corresponding International Classification of Diseases, 9th edition. The codes pertaining to a specified disease were determined using the grouping provided by Clinical Classification Software developed as part of the Healthcare Cost and Utility Project [12]. Disease prevalence is defined as the number of unique patients in each county that have a claim related to a given disease divided by the total population of the county. The average of those county-level prevalences was used as the state prevalence for each disease.

#### Adjusted Message Counts and Correction Factors

Due to ambiguity in some of the disease lexica, the message counts for each disease need to be scaled to reflect that many uses of terms such as “heart attack” or “stroke” are metaphorical or refer to other subjects such as golf “stroke.” The scaling is accomplished via a correction factor based on the manual review of tweets by 2 researchers using the methods outlined in Weeg, et al [13].

**Figure 1:**
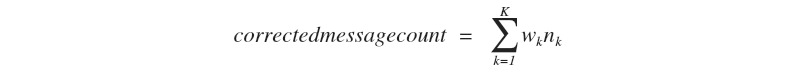
Equation for deriving a disease's corrected message count.

To calculate the correction factor for a disease, a sample of 30 tweets for each keyword were sampled. Those tweets were then classified as being a reference to a disease or not a reference to a disease. The percentage of tweets from the sample pertaining to a disease was identified as the correction factor for that keyword, *w*_*k*_. To calculate the corrected message count for a disease (Figure 1), the product of the correction factor, *w*_*k*_, and the number of messages containing that keyword, *n*_*k*_, are summed for all keywords for a single disease.

#### Comparing Tweet Volume to Disease Prevalence in Pennsylvania

We used summary statistics to compare the volume of posts on Twitter with the disease prevalence in Pennsylvania for those conditions.

#### Associating Disease with Themes

**Figure 2:**
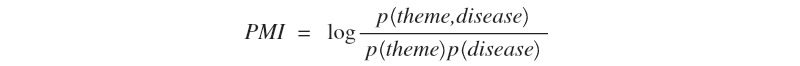
Equation for deriving the pointwise mutual information between a disease and a theme. PMI: pointwise mutual information.

The distribution of themes was investigated using 2 different metrics: the probability of the theme given the disease and the pointwise mutual information (PMI) between the disease and theme (Figure 2). The probability of the theme given the disease provides insight into the most prevalent topics of conversation for the given disease.

The PMI of a disease and theme provides a measure of how often a disease and theme co-occur relative to how often the 2 would co-occur if independent of one another. This provides insight into theme-disease co-occurrence that may be somewhat rare but is significantly different from random chance.

## Results

### Tweet Volume and Disease Prevalence Comparison

#### Tweet Volume

The initial sample of tweets from Pennsylvania consisted of 408,296,620 tweets. The data were filtered for messages containing disease-related language, resulting in a dataset containing 226,802 messages. This estimated size of this dataset was further reduced to 174,381 messages after correction factors were applied to the disease message counts. Breast cancer (n=39,156), stroke (n=53,858), and diabetes (n=41,615) were the most frequent conditions represented in the dataset (Table 1).

#### Correction Factors and Corrected Message Counts

Of the 14 diseases, we identified only 2, COPD and stroke, with a correction factor below 90% (Table 1). Messages containing terms related to pancreatic and ovarian cancer were always a direct reference to the disease. References to stroke were nonmedical or references to other health topics, such as heat stroke, 84.88% (45,716/53,858 messages) of the time.

#### Comparing Tweet Volume to Disease Prevalence in Pennsylvania

**Figure 3:**
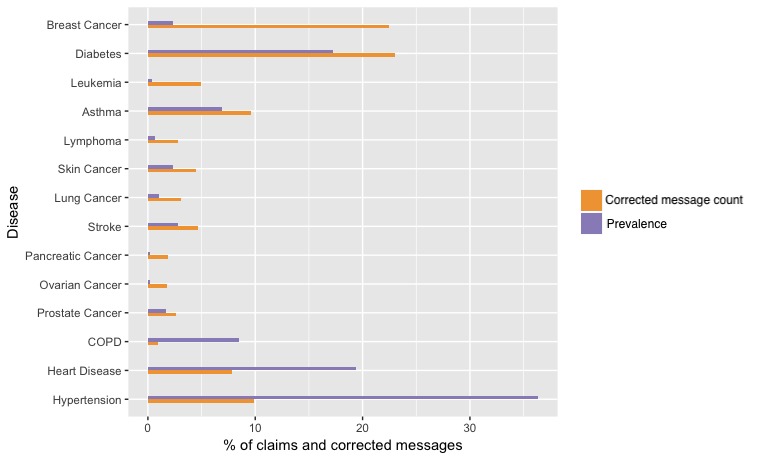
Proportion of messages versus prevalence. COPD: chronic obstructive pulmonary disease.

When comparing prevalence to corrected message counts (Figure 3) we identified that hypertension (17,245/174,381 messages, 9.89%; 4614,776/12,702,379 prevalence, 36.33%), COPD (1648/174,381 messages, 0.95%; 1,083,627/12,702,379 prevalence, 8.53%), and heart disease (13,669/174,381 messages, 7.84%; 2,461,721/12,702,379 prevalence, 19.38%) were underrepresented on Twitter. Breast cancer was overrepresented when comparing corrected message counts and prevalence (39,156/174,381 messages, 22.45%; 306,127/12,702,379 prevalence, 2.41%).

**Table 1:** Characteristics of the study sample: tweet data and user data.

Disease		Message count, n	Correction factor, %	Corrected message count, n	Users, n
**Cancer**					
	Breast cancer	39,169	100	39,156	19,960
	Leukemia	9129	95.1	8682	5855
	Lung cancer	5745	92.6	5317	3719
	Lymphoma	5276	93.4	4927	2758
	Ovarian cancer	3063	99.9	3060	1212
	Pancreatic cancer	3231	100	3231	1189
	Prostate cancer	4487	100	4487	2311
	Skin cancer	7866	99.9	7859	4048
**Chronic lung disease**					
	Asthma	18,082	92.6	16,742	10,185
	Chronic obstructive pulmonary disease	2137	77.1	1648	726
	Diabetes	41,615	96.6	40,217	16,321
**Heart disease**					
	Heart disease	14,740	92.7	13,669	7992
	Hypertension	18,404	93.7	17,245	12,203
Stroke		53,858	15.1	8141	34,298

### Characterizing Tweet Topics About Individual Diseases

 For each disease, we identified all statistically significant (*P*<.001) correlations between topics and a binary label indicating whether or not a message contained a reference to the disease. Topics most correlated with asthma were related to first-person accounts of managing the disease (*attack* and *inhaler*), discomfort associated with the disease (*can’t* and* breathe*), or conditions that pose additional risk *(pollution, mold,* and *dust)* such as allergens. The majority of topics associated with cancer referenced some variety of charity campaign (*pink, ribbon,* and *bracelet*) or awareness effort (*support, awareness, October,* and *pink*). Topics related to stroke were rarely related to cerebrovascular accident, but more often related to other definitions of stroke (eg, golf stroke, paint stroke, and heat stroke). Diabetes, heart disease, and hypertension messages were correlated with topics that focused on disease management (*weight loss, insulin,* and *reduce stress*) and lifestyle choices (*diet* and *exercise)*. Complete topic word clouds for each disease can be found in Multimedia Appendix 2.

### Characterizing Tweet Themes Across Diseases

#### Probability of Theme Given Disease

**Figure 4:**
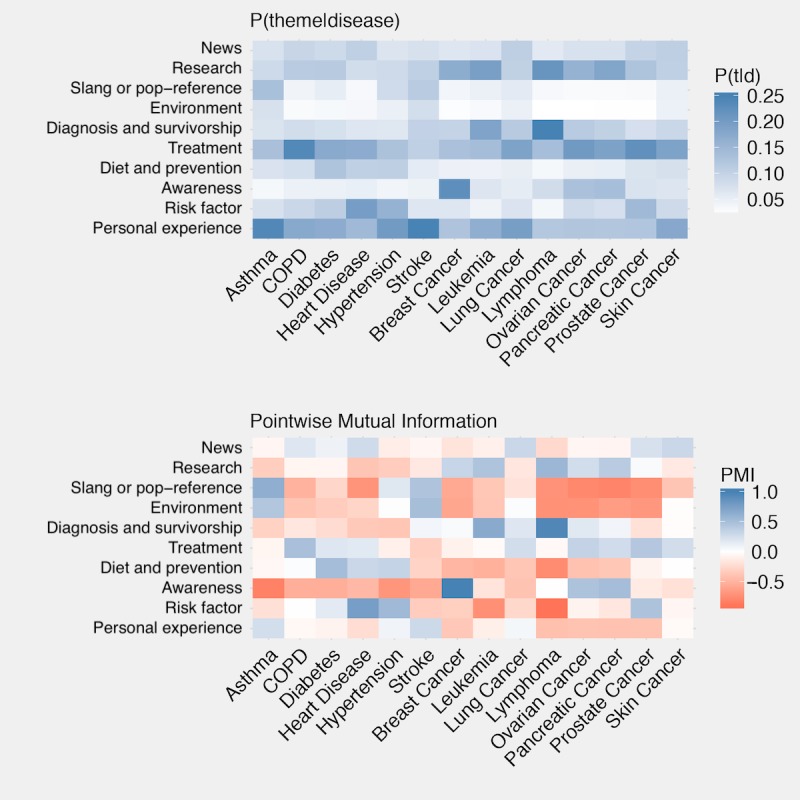
Theme distribution. P(t|d): probability of theme given disease; COPD: chronic obstructive pulmonary disease; PMI: pointwise mutual information.

The probability of a theme given the disease provides insight into the most prevalent topics of conversation for a specific disease (Figure 4). We identified that messages referencing breast cancer were more likely to be about disease *awareness* (9139/39,156 messages, 23.34%). Heart disease messages mostly focused on *risk factors* such as stress, sleep, and obesity (1375/13,669 messages, 10.06%). In most cases, asthma messages referenced a *personal experience*.

#### Pointwise Mutual Information

PMI provides a measure of association between the theme and the disease (Figure 4). We found that diagnosis was a small proportion of the theme distribution for each disease. However, if diagnosis or survivorship is mentioned, it is much more likely to be mentioned in conjunction with lymphoma and leukemia than with the other diseases (PMI 0.67-0.96). Similarly, a relationship between the *risk factors* theme and hypertension and heart disease was found (PMI 0.54-0.77).

## Discussion

### Principal Findings

There is increasing focus on the potential for big data from digital sources in health care. There are challenges associated with using these sources, as they are not always collected for the purposes of health tracking.

We explored the potential for using Twitter to better understand the Web-based conversation about common health conditions. We identified that in some cases, traditional health metrics are associated with the volume of tweets for a given disease. Although traditional methods of determining disease prevalence are robust, they are often delayed in availability because the process for data acquisition and tracking to determine reliable and valid estimates is considerable. Twitter data are available in real-time, much faster than traditional methods, and with significant volume providing a measure of public discourse about health. While tweets would not replace traditional surveillance in the way initially posed by Google flu trends [14], they do provide something unique that prevalence statistics do not; a narrative about patient and public thoughts, knowledge, and experiences with health. Twitter provides context to the conversation surrounding disease and allows for characterization of public discussion of high prevalence conditions. We identified that individuals are using Twitter to talk about several diseases, although variation exists in the frequency of disease mention and the content.

We observed that people are using Twitter for talking about the most common health conditions in Pennsylvania. Prior work has demonstrated the use of Twitter to monitor influenza [15], postpartum depression [16], concussion [17], epilepsy [18], and migraine [19]. The prevalence of disease has been correlated with the frequency of Twitter posting across a variety of diseases [13,20].

We also identified variability in disease mentions and the specificity of terms. This finding provides us with several insights. First, heart disease and stroke cannot be analyzed without preprocessing owing to the ambiguity of many of the keywords associated with the diseases. To resolve these varying issues, other methods will need to be developed to filter out much of the noise associated with these diseases. However, this finding also assures us that the majority of the language we find associated with other diseases can be analyzed using the open vocabulary methods previously described with minimal preprocessing.

Although disease prevalence often coincides with disease mention on Twitter, we found significant variability. The frequency of mentions of breast cancer on Twitter was several orders of magnitude higher than lung cancer, although lung cancer has a higher rate of death and relatively similar prevalence. Breast cancer has a large social media presence owing to awareness and charity campaigns in conjunction with a large community base from those affected by the disease. Lung cancer is tweeted about less often and is often the result of a pop culture reference from television or a celebrity death.

Traditional metrics provide detailed information about prevalence but not insights about people’s understanding, concerns, and questions about health and disease. Our analysis identified several underlying themes that are specific to some diseases. Asthma tweets included references to personal experiences for both the person with asthma as well as parents expressing concern for their children’s asthma issues. Although the largest portion of tweets for the different types of cancer analyzed often referenced charity and awareness, we observed that across diseases in our sample, cancer conditions had the largest portion of tweets about diagnosis.

Our findings also give insight into potential opportunities for using Twitter to inform public health and health communications practices. Future work could examine temporal relationships between Twitter volume and semantic data and traditional health data over larger timeframes and at varying timescales. Meaningful temporal relationships may indicate that Twitter data have value as an additional signal to augment existing surveillance systems, allowing for more precise health tracking and timely interventions.

Twitter data could enhance community building and engagement. Prior work by Neiger et al [21,22] found that more two-way communication on Twitter between public health entities and individual citizens led to an increase in action and awareness that, in turn, resulted in an improvement in community health. Providing local and state public health entities with more accurate information on the public discourse surrounding health could enhance communication and contribute to the more effective dissemination of pertinent and timely health information to the public.

Finally, understanding the interaction between social media use and individual health can identify opportunities for targeted interventions. Prior work by Park et al [23] showed that interventions targeting the perception of social media interaction have the potential to positively impact individual health. We have shown that it is possible to capture a measure of public perception of individual diseases at the community level via analysis of topics and themes. These methods can be translated to individual subjects, where disease perceptions could be tracked over time and compared with actual measures of health, potentially identifying opportunities for intervention.

### Limitations

We compared data from Twitter for 2012-2015 with disease prevalence from 2014, so there may be some variability by year in these estimates. We evaluated unadjusted data from 1 state, so this may not be representative of the conversation about health conditions across other states or geographic regions. Twitter data primarily originate from urban areas; hence, data may not be the most representative sample across the state of Pennsylvania. Future work could explore variations in language on Twitter relative to the size of geographic regions, socioeconomic factors (eg, race, income, urban or rural), and variations in news events or other triggers. Although our correction method eliminates nondisease references, it does not account for metaphorical and joking tweets. This impacts diseases such as heart disease, diabetes, and hypertension.

The precision of the disease keyword filtering, which is the number of selected tweets that were relevant, is reasonably estimated by the corrected message count. However, the recall of the disease keyword filtering, which is the number of relevant tweets that were selected, is difficult to determine owing to the nature of the data and the subjectivity of relevance in the context of health-related tweets. Hopkins et al [24] provides 3 different models for estimating recall: a hand-coding approach similar to the corrected message count presented here, a supervised learning approach for individual document classification, and a supervised learning approach to estimate document category proportions. Evaluating these methods in terms of cost and accuracy is beyond the scope of this study but should be considered for future work to provide more robust measures of keyword-filtered data quality.

Location identification accuracy is difficult to measure for user-defined locations owing to the relative ambiguity of the data provided. The procedures used to estimate user-defined location provide a “soft” measure of accuracy, but more work is needed to ensure appropriate representation. Additionally, a very small proportion of tweets contains location information, thus, the sample may not be representative of the general Twitter landscape in Pennsylvania. Methods such as those detailed in Liang et al [25] should be considered in future studies to correct for sampling bias.

### Conclusions

We identified that the volume of tweets is often related to rates of health conditions across a state. The semantic content provided from Twitter provides insight into public perception and awareness of disease beyond what is available through traditional measures of disease prevalence.

## Appendix

Multimedia Appendix 1 Consumer Health Vocabulary search terms: This study focuses on 14 diseases and each disease is represented by a lexicon of disease related terms. The appendix contains each of the 14 diseases along with the 274 terms which comprise the lexica.

Multimedia Appendix 2 Correlation between topic and disease.

## Abbreviations

API: application programming interface
COPD: chronic obstructive pulmonary disease
LDA: latent Dirichlet allocation
PMI: pointwise mutual information

## References

[1] Prieto VM, Matos S, Álvarez M, Cacheda F, Oliveira JL (2014). Twitter: a good place to detect health conditions. PLoS One.

[2] Eysenbach G (2009). Infodemiology and infoveillance: framework for an emerging set of public health informatics methods to analyze search, communication and publication behavior on the Internet. J Med Internet Res.

[3] Laranjo L, Arguel A, Neves AL, Gallagher AM, Kaplan R, Mortimer N, Mendes GA, Lau AYS (2015). The influence of social networking sites on health behavior change: a systematic review and meta-analysis. J Am Med Inform Assoc.

[4] Wehner Mackenzie R, Chren Mary-Margaret, Shive Melissa L, Resneck Jack S, Pagoto Sherry, Seidenberg Andrew B, Linos Eleni (2014). Twitter: an opportunity for public health campaigns. Lancet.

[5] Lee JL, DeCamp M, Dredze M, Chisolm MS, Berger ZD (2014). What are health-related users tweeting? A qualitative content analysis of health-related users and their messages on twitter. J Med Internet Res.

[6] Hill S, Merchant R, Ungar L (2013). Lessons Learned About Public Health From Online Crowd Surveillance. Big Data.

[7] Eichstaedt JC, Schwartz HA, Kern ML, Park G, Labarthe DR, Merchant RM, Jha S, Agrawal M, Dziurzynski LA, Sap M, Weeg C, Larson EE, Ungar LH, Seligman MEP (2015). Psychological language on Twitter predicts county-level heart disease mortality. Psychol Sci.

[8] Preotiuc-Pietro D, Samangooei S (2012). Trendminer: An architecture for real time analysis of social media text. http://www.aaai.org/ocs/index.php/ICWSM/ICWSM12/paper/download/4739/5087.

[9] Collaborative Consumer Health Vocabulary Initiative, Biomedical Informatics Department University of Utah (2018). Consumer Health Vocabulary Initiative.

[10] Schwartz H, Eichstaedt J, Kern M, Dziurzynski L, Lucas R, Agrawal M, Park G, Lakshmikanth S, Jha S, Seligman M, Ungar L (2013). Characterizing geographic variation in well-being using tweets.

[11] Schwartz HA, Eichstaedt JC, Kern ML, Dziurzynski L, Ramones SM, Agrawal M, Shah A, Kosinski M, Stillwell D, Seligman MEP, Ungar LH (2013). Personality, gender, and age in the language of social media: the open-vocabulary approach. PLoS One.

[12] Healthcare Cost and Utilization Project (HCUP).

[13] Weeg C, Schwartz HA, Hill S, Merchant RM, Arango C, Ungar L (2015). Using Twitter to Measure Public Discussion of Diseases: A Case Study. JMIR Public Health Surveill.

[14] Ginsberg J, Mohebbi MH, Patel RS, Brammer L, Smolinski MS, Brilliant L (2009). Detecting influenza epidemics using search engine query data. Nature.

[15] Paul M, Dredze M (2011). You are what you Tweet: Analyzing Twitter for public health.

[16] De Choudhury M, Counts S, Horvitz E (2013). Predicting postpartum changes in emotion and behavior via social media.

[17] Sullivan SJ, Schneiders AG, Cheang C, Kitto E, Lee H, Redhead J, Ward S, Ahmed OH, McCrory PR (2012). 'What's happening?' A content analysis of concussion-related traffic on Twitter. Br J Sports Med.

[18] McNeil K, Brna PM, Gordon KE (2012). Epilepsy in the Twitter era: a need to re-tweet the way we think about seizures. Epilepsy Behav.

[19] Nascimento TD, DosSantos MF, Danciu T, DeBoer M, van Holsbeeck H, Lucas SR, Aiello C, Khatib L, Bender MA, UMSo DCO2, Zubieta J, DaSilva AF (2014). Real-time sharing and expression of migraine headache suffering on Twitter: a cross-sectional infodemiology study. J Med Internet Res.

[20] Young SD, Rivers C, Lewis B (2014). Methods of using real-time social media technologies for detection and remote monitoring of HIV outcomes. Prev Med.

[21] Neiger BL, Thackeray R, Burton SH, Thackeray CR, Reese JH (2013). Use of twitter among local health departments: an analysis of information sharing, engagement, and action. J Med Internet Res.

[22] Thackeray R, Neiger BL, Burton SH, Thackeray CR (2013). Analysis of the purpose of state health departments' tweets: information sharing, engagement, and action. J Med Internet Res.

[23] Park J, Lee DS, Shablack H, Verduyn P, Deldin P, Ybarra O, Jonides J, Kross E (2016). When perceptions defy reality: The relationships between depression and actual and perceived Facebook social support. J Affect Disord.

[24] Hopkins D, King G (2010). A Method of Automated Nonparametric Content Analysis for Social Science. Am J Political Sci.

[25] Liang H, Shen F, Fu K (2016). Privacy protection and self-disclosure across societies: A study of global Twitter users. New Media & Society.

