# Supplementary figures and images for: Characterizing Tweet Volume and Content About Common Health Conditions Across Pennsylvania: Retrospective Analysis
Source: JMIR Public Health Surveill. 2018 Dec 6;4(4):e10834. doi: 10.2196/10834 (PMC6302232; doi:10.2196/10834)

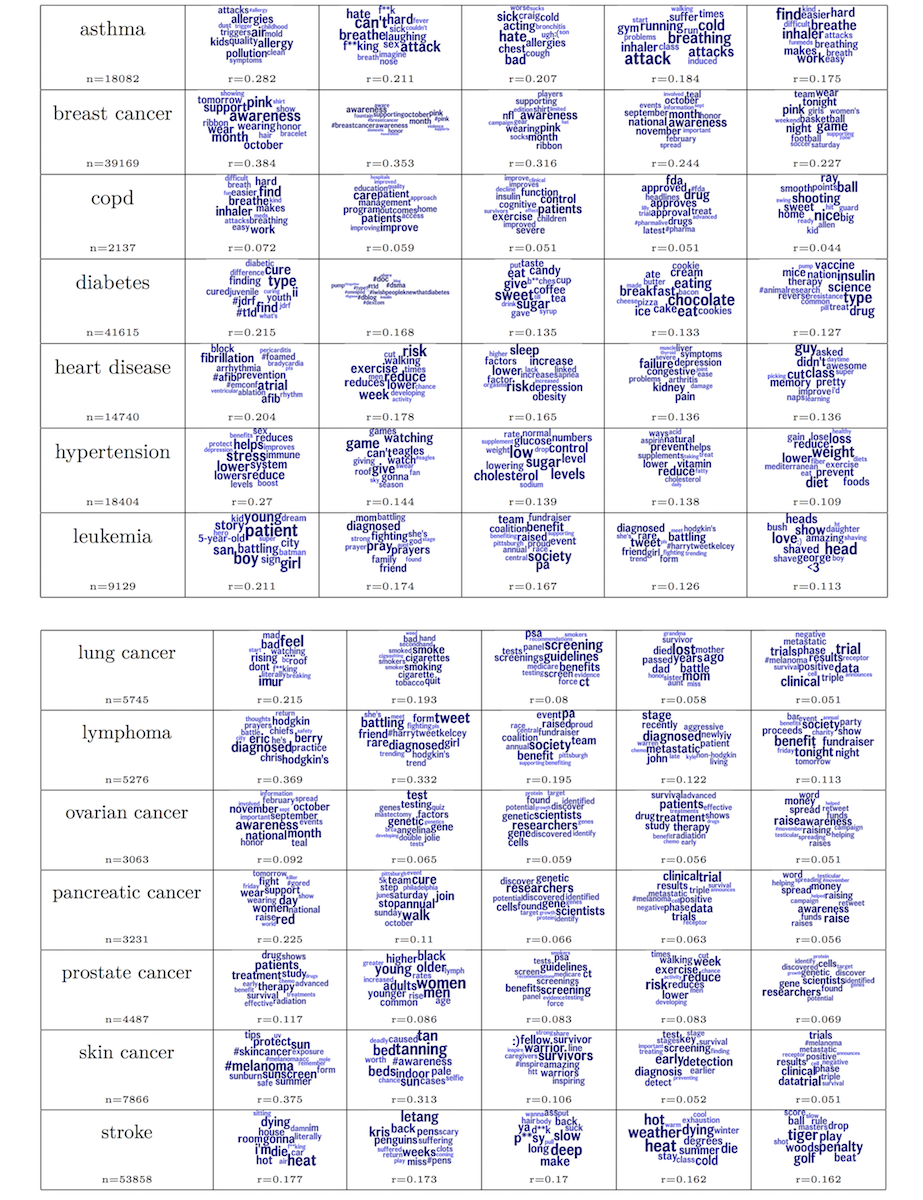

Supplement: Multimedia Appendix 2 [file publichealth_v4i4e10834_app2.png]
